# Beyond mutation burden: biological termination as a systems perspective on cancer

**DOI:** 10.3389/fcell.2026.1942325

**Published:** 2026-10-13

**Authors:** Ivan Shterev Donev, Mila Petrova Petrova, Mariyana Ilieva Eneva, Yavor Kostadinov Kashlov, Sonya Draganova

**Affiliations:** 1 Clinic of Medical Oncology, MHAT “Nadezhda”, Sofia, Bulgaria; 2 Hospital Pharmacy, MHAT “Nadezhda”, Sofia, Bulgaria; 3 Department of Propedeutics of Internal Diseases, UMHAT “St. Marina”, Varna, Bulgaria

**Keywords:** biological termination, cancer biology, carcinogenesis, gene regulatory networks, information theory

## Abstract

Cancer is traditionally viewed as a genetic disease driven by the accumulation of mutations that disrupt normal cellular regulation. While this framework has generated profound biological insights, several observations remain incompletely explained, including the presence of driver mutations in normal tissues, the occurrence of highly aggressive tumors with low mutational burdens, and the convergence of genetically diverse cancers toward similar phenotypic states. This article proposes that malignant transformation may be understood as a progressive failure of biological termination mechanisms responsible for constraining developmental, proliferative, and adaptive cellular programs. Drawing upon concepts from information theory, systems biology, developmental biology, and regulatory network dynamics, cancer is interpreted as a persistent pathological state in which biological processes that would normally terminate remain active beyond their physiological context. This framework generates several testable predictions, including the emergence of aggressive tumors despite limited mutational complexity, convergence of distinct genetic alterations upon common regulatory attractors, and the potential prognostic value of measures of informational and regulatory disorganization. Together, these observations suggest that malignant behavior may depend not only on genetic alterations themselves but also on the organizational states through which biological information is interpreted and executed.

## Entropy and information: two complementary dimensions of biological organization

The Second Law of Thermodynamics states that entropy, a measure of disorder within a physical system, increases over time. Living organisms maintain highly ordered cellular and molecular structures by continuously expending energy to counteract this tendency. Genomic integrity, gene regulation, protein homeostasis, and tissue architecture all depend on the sustained maintenance of biological order.

Biological order is determined not only by physical organization but also by the integrity of information storage and processing (Noble, 2012). Cellular identity depends upon the accurate preservation, transmission, and interpretation of genetic and epigenetic information.

Cancer has traditionally been interpreted as a consequence of genetic abnormalities and dysregulated biochemical pathways (Hanahan and Weinberg, 2011). However, cellular behavior emerges from the integration of genomic, epigenetic, metabolic, and environmental information across multiple levels of biological organization. Consequently, malignant phenotypes cannot always be explained by simple linear relationships between individual molecular alterations and biological outcomes but instead arise from complex interactions among regulatory networks operating within the cell and its microenvironment.

Claude Shannon’s theory of information provides a quantitative framework for describing uncertainty within complex systems (Shannon, 1948). In this context, informational entropy increases as uncertainty rises and predictability decreases. Applied to biology, mutations, replication errors, and structural genomic alterations reduce the fidelity with which genetic information can be stored, transmitted, and executed, thereby increasing uncertainty within the system.

Maintenance of genomic fidelity depends on multiple DNA repair pathways, among which the mismatch repair (MMR) system plays a central role (Baretti and Le, 2018). By correcting replication-associated mismatches, MMR preserves genomic stability and limits the accumulation of mutations. Deficiency of this system results in microsatellite instability and progressive mutational accumulation, increasing genomic uncertainty and facilitating malignant adaptation.

Although only approximately 1%–2% of the human genome encodes proteins, many characterized non-coding elements have important roles in gene regulation, chromatin organization, and higher-order genome architecture (Dunham et al., 2012). These characterized regulatory elements contribute to cellular differentiation, developmental programming, and adaptive responses to environmental stimuli, while the functions of a substantial fraction of non-coding sequence remain incompletely characterized. Consequently, biological function depends not only on genetic content but also on the regulatory architecture governing the interpretation and execution of that content.

Alterations within regulatory sequences can exert substantial biological effects despite leaving protein-coding regions unchanged. Regulatory elements located within untranslated regions, enhancers, silencers, and chromatin-associated domains influence gene expression, protein activity, and cellular behavior, illustrating that biological information is distributed across both coding and non-coding components of the genome (Luo et al., 2025).

Genome topology also contributes directly to gene regulation. Structures such as G-quadruplexes influence transcription, replication, and chromatin organization, demonstrating that biological information is encoded not only in nucleotide sequence but also in higher-order structural configurations that regulate access to and interpretation of genetic information (Hänsel-Hertsch et al., 2017).

## Computation, information, and biological organization

Several concepts from theoretical computer science provide useful analogies for understanding biological systems (Grozinger et al., 2025). The Curry–Howard correspondence established a formal relationship between logic and computation, demonstrating that abstract logical statements can be represented as executable computational processes (Howard, 1986). Subsequent work by Lambek extended these ideas to category theory, providing a mathematical framework for describing transformations between structured systems. Although biological systems are not digital computers, these developments highlight a broader principle: complex behaviors can emerge from the execution of organized information (Grozinger et al., 2025).

This perspective has influenced computational approaches to biology, including membrane computing and abstract chemistry, which model biological organization as the interaction of information-processing entities within structured environments (Grozinger et al., 2025). Together, these frameworks emphasize that biological behavior depends not only on molecular components but also on the rules governing their interactions.

In living systems, genetic information is continuously interpreted through regulatory networks coordinating development, differentiation, adaptation, and tissue maintenance (Noble, 2012). Cellular behavior therefore emerges not simply from the presence of genes or proteins but from the execution of organized informational processes operating across multiple levels of biological organization. From this perspective, malignant transformation may be viewed as a disruption of the mechanisms that normally constrain and terminate such processes.

## Biological information processing and program termination

DNA represents a highly organized biological information system in which genetic instructions are encoded through a finite nucleotide alphabet and executed through coordinated molecular processes including replication, transcription, and translation (Blackmond, 2010). Cellular behavior depends not only on the content of these instructions but also on the regulatory mechanisms governing when, where, and for how long biological programs remain active.

The genome therefore functions within a broader informational architecture that includes chromatin organization, epigenetic regulation, signaling pathways, metabolic networks, and interactions with the cellular microenvironment. These systems collectively determine whether developmental, proliferative, adaptive, and survival programs are initiated, maintained, or terminated.

Experimental observations suggest that malignant behavior cannot be explained solely by alterations in DNA sequence. In the 1970s, Mintz and Illmensee demonstrated that malignant teratocarcinoma cells retained the capacity to participate in normal embryonic development when introduced into mouse blastocysts (Mintz and Illmensee, 1975). Tumor-derived cells contributed to differentiated tissues within the developing embryo, demonstrating context-dependent developmental plasticity and suppression of malignant phenotype under embryonic regulatory conditions. Because the experiment predates modern genomic characterization, it does not establish that the tumor-derived cells were genetically normal.

Subsequent nuclear-transfer experiments produced heterogeneous results across tumor models. Nuclei from leukemia, lymphoma, and breast cancer models could support aspects of pre-implantation development but did not generate embryonic stem cells, whereas an inducible RAS melanoma model demonstrated greater reprogramming potential, including generation of embryonic stem cells with differentiation capacity (Hochedlinger et al., 2004). These findings are best interpreted as evidence of context-dependent developmental plasticity rather than evidence that tumor genomes are intrinsically normal. Conversely, cybrid studies revealed that extranuclear factors, including mitochondrial and metabolic networks, can substantially influence tumorigenic potential even in the presence of an unchanged nuclear genome (Israel and Schaeffer, 1988).

Collectively, these observations challenge a purely gene-centric interpretation of cancer and suggest that malignant behavior emerges from dynamic interactions among genomic, cytoplasmic, metabolic, and microenvironmental regulatory systems. Cellular phenotype therefore depends not only on genetic information itself but also on the biological context in which that information is interpreted and executed (Hochedlinger et al., 2004).

## The Halting Problem as a conceptual analogy

The parallels between biological information processing and computation have inspired comparisons with concepts originating in theoretical computer science. Among the most influential is Alan Turing’s Halting Problem, which demonstrated that no universal algorithm can determine whether every possible computation will eventually terminate or continue indefinitely (Grozinger et al., 2025).

Biological systems are not formal computational systems in the mathematical sense, and the Halting Problem should therefore be regarded as a conceptual analogy rather than a literal biological mechanism. The application of computational concepts to biology is supported by theoretical work demonstrating that biochemical reaction networks can, under appropriate conditions, perform universal computation (Grozinger et al., 2025). Cellular regulatory systems process information, integrate environmental signals, execute conditional responses, and coordinate complex state transitions through distributed molecular interactions. Although living cells are not digital computers, these observations suggest that biological processes may be analyzed using concepts developed in computation theory, including information processing, state transitions, and program termination (Fages et al., 2017), (Chen et al., 2014), (Hjelmfelt et al., 1991), (Feret and Koeppl, 2017). Nevertheless, it highlights an important principle: in sufficiently complex systems, long-term behavior may be difficult to predict solely from the instructions from which the system originates (Grozinger et al., 2025).

Cells continuously execute regulatory programs governing proliferation, differentiation, survival, repair, migration, and adaptation. Under physiological conditions, these programs are activated transiently and terminated once their biological function has been fulfilled. Tissue repair ceases following wound closure, developmental programs terminate after organ formation, and cells carrying irreparable damage undergo senescence or apoptosis.

The maintenance of organismal homeostasis therefore depends upon mechanisms capable of restricting, silencing, eliminating, or otherwise terminating biological programs that are no longer required. Failure of these mechanisms can permit the persistence of cellular states that would normally be transient.

## Definition of biological termination

For the purposes of this framework, biological termination refers to the coordinated execution of mechanisms that arrest, eliminate, differentiate, silence, or otherwise deactivate a biological program once its physiological role has been completed. These mechanisms include cell-cycle checkpoints, differentiation pathways, apoptosis, senescence, immune surveillance, developmental control systems, and tissue-level regulatory feedback loops (Han and ahan, 2022).

Successful termination ensures that biological processes remain temporally and spatially constrained. Failure of termination allows self-maintaining cellular states to persist beyond their normal context.

## Cancer as failure of biological termination

Within this framework, cancer may be interpreted as a progressive failure of biological termination. The defining characteristic of the malignant state is not merely increased proliferation but the inability of cellular systems to appropriately terminate proliferative, developmental, adaptive, and survival programs.

Genetic mutations, epigenetic alterations, chromatin remodeling, mitochondrial dysfunction, and microenvironmental changes may all contribute to this process. However, these factors are viewed not as independent causes of malignancy but as alternative mechanisms through which normal termination systems become compromised.

The consequence is the emergence of persistent regulatory states characterized by continued proliferation, resistance to elimination, impaired differentiation, adaptive plasticity, and evolutionary persistence despite signals that would ordinarily restrict cellular expansion.

## Gestational trophoblastic disease: a model of malignant behaviour beyond classical somatic mutation

Gestational trophoblastic disease (GTD), particularly complete hydatidiform moles and choriocarcinoma, provides a unique model for examining the relationship between genomic organization, developmental regulation, and malignant behavior (Bahutair et al., 2024). While contemporary models of oncogenesis emphasize the accumulation of somatic mutations, GTD demonstrates that aggressive proliferative phenotypes may arise through profound disturbances of developmental control and genomic imprinting.

In complete hydatidiform moles, an enucleated ovum is fertilized by one or more spermatozoa, resulting in a conceptus containing an almost exclusively paternal genome (Bahutair et al., 2024). Despite the absence of the extensive mutational burden typically associated with many adult cancers, these lesions exhibit uncontrolled trophoblastic proliferation and, in some cases, progression to invasive disease or choriocarcinoma.

Normal embryonic development depends upon coordinated interactions between maternally and paternally derived genetic programs. Through genomic imprinting, these parental contributions regulate growth, differentiation, trophoblastic invasion, and developmental timing. In GTD, disruption of this balance permits physiological trophoblastic programs to persist beyond their normal developmental context.

The significance of GTD extends beyond its clinical manifestations. It demonstrates that aggressive proliferative behavior can occur without the extensive somatic mutational burden characteristic of many adult cancers, despite profound underlying genomic and developmental abnormalities. From the perspective proposed here, GTD can be interpreted as an example of disrupted developmental program termination rather than as a phenotype explained primarily by extensive somatic mutational accumulation.

## Cancer-associated mutations without cancer

The widespread presence of cancer-associated mutations in histologically normal tissues suggests that malignant transformation cannot be explained solely by the accumulation of genetic alterations. Numerous studies have demonstrated that driver mutations and clonal expansions are common features of normal aging tissues, yet only a small fraction of these cellular populations ultimately progress to cancer (Kennedy et al., 2019), (Martincorena, 2019).

These observations imply that the presence of oncogenic mutations alone is insufficient to generate malignant behavior and that additional factors governing tissue organization, regulatory network integrity, and cellular state may play critical roles in determining whether a mutated clone remains benign or undergoes malignant transformation.

From this perspective, carcinogenesis may depend not only on the acquisition of mutations but also on the emergence of permissive biological states capable of sustaining and stabilizing malignant programs.

The presence of canonical oncogenic driver mutations is not, by itself, sufficient to induce malignant transformation in primary human cells (Hahn et al., 1999), (Akagi et al., 2003). Activating alterations in genes such as EGFR and KRAS have been identified in histologically normal lung tissue, indicating that oncogenic mutations may persist without producing overt cancer (Hill et al., 2023), (Chalela et al., 2019). Large-scale efforts such as the Somatic Mosaicism across Human Tissues (SMaHT) programme further emphasize the importance of characterizing somatic genetic variation in histologically normal tissues and may help clarify why some altered cellular states remain constrained while others progress toward malignancy (SMaHT Program, NIH, 2024).

These findings support the view that carcinogenesis depends not only on driver mutations themselves but also on the biological context in which such alterations operate, including tissue organization, cellular state, and microenvironmental influences.

## Predictions derived from the biological termination failure framework

The central premise of the present framework is that malignant transformation reflects a progressive failure of biological termination mechanisms responsible for constraining developmental, proliferative, and adaptive cellular programs. Under this model, genetic mutations, epigenetic alterations, chromatin remodeling, and microenvironmental influences represent alternative pathways through which normal regulatory termination may be disrupted. Several experimentally testable predictions arise from this interpretation.

## Prediction 1: highly aggressive tumours may arise despite low mutational burden

Although increasing genetic complexity may accompany tumour evolution, malignant aggressiveness is not linearly determined by the number of somatic alterations. The present framework therefore predicts that extensive mutational accumulation is not a necessary prerequisite for malignant behaviour.

The low mutational burden observed in many pediatric malignancies is consistent with the hypothesis that oncogenesis may arise through failure of developmental program termination, whereby embryonic or lineage-specific regulatory states persist beyond their normal physiological context and acquire malignant characteristics (Chen et al., 2024). Similarly, gestational trophoblastic disease demonstrates that highly aggressive biological behavior can emerge without an extensive somatic mutational burden, despite profound genomic and developmental abnormalities, when developmental regulatory systems become profoundly dysregulated.

From a systems perspective, malignant behavior may depend not only on the quantity of genomic alterations but also on their impact on regulatory architecture and information flow. Aggressiveness may therefore emerge from disruption of network organization rather than mutational burden itself.

The limitations of tumor mutational burden (TMB) as a predictive biomarker further support this interpretation. Tumors with similar mutational burdens often exhibit markedly different clinical behaviors and therapeutic responses, suggesting that the organization of developmental programs, regulatory networks, and biological information processing may be more important determinants of malignant potential than mutation counts alone (Gurjao et al., 2023), (Jardim et al., 2021).

Consequently, tumor aggressiveness may depend more strongly on disruption of developmental and regulatory architecture than on the absolute number of mutations present within the genome.

## Prediction 2: distinct genetic alterations should converge upon common non-terminating biological states

Emerging evidence suggests that genetically diverse cancers may converge upon a limited number of common regulatory states. Pan-cancer analyses have identified recurrent gene-expression attractors that appear across multiple tumour types despite substantial differences in mutational profiles, while systems-level models indicate that advanced tumours occupy similar regions within the gene-expression landscape independently of patient age or genetic background (Cheng et al., 2013), (Gonzalez et al., 2023).

Such observations support the view that malignant progression reflects movement toward stable network states characterized by persistent self-maintenance, impaired differentiation, resistance to apoptosis, and continued proliferation. From this perspective, mutations affecting tumour suppressors, oncogenic pathways, developmental regulators, chromatin modifiers, or DNA repair systems may represent alternative routes leading to the same pathological attractor.

The phenomenon of epithelial-to-mesenchymal transition (EMT) further illustrates how distinct molecular alterations may converge upon common regulatory states. Diverse genetic, epigenetic, and microenvironmental perturbations can induce remarkably similar transcriptional programs characterized by increased cellular plasticity, invasiveness, therapeutic resistance, and metastatic potential (Pastushenko and Blanpain, 2019). Conversely, successful metastatic colonization often requires partial or complete mesenchymal-to-epithelial transition (MET), demonstrating that tumour progression depends upon transitions between a limited number of dynamically stable regulatory states rather than on specific mutations alone.

These observations suggest that the critical determinant of malignant behaviour may reside not exclusively in the initiating genetic lesion but in the emergent regulatory state toward which the biological system evolves.

## Prediction 3: measures of informational and regulatory disorganization may predict clinical behaviour better than mutation counts alone

Current genomic approaches frequently rely on mutational burden, driver mutations, and genomic instability as indicators of tumour behaviour. However, growing evidence suggests that measures of biological information processing may provide complementary or even superior insights into malignant potential.

Cellular function emerges from the coordinated activity of regulatory networks operating across multiple levels of organization, including chromatin architecture, gene expression programs, signalling pathways, metabolic systems, and cell-to-cell interactions (Han and ahan, 2022). Consequently, tumours with comparable mutational burdens may exhibit markedly different clinical behaviours depending on the degree of regulatory disorganization present within these networks.

Studies employing entropy-based approaches have demonstrated associations between signalling complexity, network entropy, tumour aggressiveness, and clinical outcome (Juarez-Flores and José, 2018), (Conforte et al., 2019). These findings support the possibility that measures of informational entropy and regulatory organization may provide complementary prognostic information beyond mutation counts alone.

Just as physical systems often evolve toward dynamically stable trajectories, biological regulatory networks may evolve toward stable attractor states. Malignancy may therefore reflect the stabilization of a pathological attractor that persists despite regulatory mechanisms that would ordinarily redirect the system toward differentiation, senescence, or elimination.

Epigenetic regulation provides a particularly compelling example of this principle. DNA methylation, histone modifications, chromatin remodeling, and higher-order chromatin organization profoundly influence gene expression without altering the underlying DNA sequence (Feinberg et al., 2016). Likewise, non-coding RNAs (lncRNAs, miRNAs, and circRNAs) and oncometabolites such as succinate and 2-hydroxyglutarate reshape regulatory networks by modifying gene expression, epigenetic states, and developmental programs (Anastasiadou et al., 2017), (Lu et al., 2012). Collectively, these observations demonstrate that malignant phenotypes may arise through disruption of biological information processing at multiple regulatory levels, independent of mutational burden alone.

If this interpretation is correct, future biomarkers derived from information theory, network topology, developmental biology, and systems-level organization may improve prediction of tumour aggressiveness, metastatic potential, therapeutic resistance, and clinical outcome.

## Discussion

The biological termination framework does not reject the importance of genetic mutations, oncogenic drivers, or clonal evolution. Rather, it proposes that these alterations acquire biological significance primarily through their effects on regulatory systems responsible for constraining, completing, or terminating cellular programs.

The widespread use of terms such as programmed cell death, developmental programs, differentiation programs, and transcriptional programs implicitly acknowledges that many biological processes can be described as organized and temporally regulated sequences of events. Within this framework, cancer may be interpreted as the pathological persistence of biological programs that normally operate only transiently. Rather than representing the emergence of entirely novel cellular behaviors, malignancy may in many cases reflect the failure of physiological programs to terminate appropriately once their biological function has been fulfilled. From this perspective, the malignant state resembles a self-maintaining biological process that continues to execute despite the loss of mechanisms that would ordinarily constrain or terminate its activity.

Several observations are consistent with this interpretation. Cancer-associated mutations are commonly detected in normal tissues without the development of overt malignancy. Certain highly aggressive tumours exhibit relatively low mutational burdens. Genetically diverse cancers frequently converge upon similar phenotypic states, and measures of network organization may predict biological behaviour more effectively than mutation counts alone.

Within this framework, mutations, epigenetic alterations, developmental dysregulation, metabolic disturbances, and microenvironmental influences are viewed as alternative mechanisms through which termination systems become compromised. Malignant transformation therefore reflects the emergence of persistent regulatory states that resist normal biological constraints.

A cell is a system in which every piece of biological information must be physically embodied in a specific chiral structure. DNA exists almost exclusively as a right-handed double helix, whereas proteins are built predominantly from left-handed amino acids (Blackmond, 2010). Consequently, biological information cannot exist merely as an abstract symbol but must be instantiated in a particular physical form. In this sense, biological existence is closer to constructive existence than to abstract logical existence: to exist biologically is to be physically realized through specific molecular structures and processes.

The remarkable convergence of discoveries in epigenetics, chromatin biology, non-coding RNAs, oncometabolites, developmental biology, and systems biology suggests that malignant transformation is increasingly understood as a disorder of regulatory organization rather than a phenomenon explained solely by mutation accumulation.

## A constructive dynamical formulation

The principal limitation of this work is that the Curry–Howard–Lambek correspondence provides a fundamentally static formal framework, whereas living systems are intrinsically dynamic. Nevertheless, it offers a useful perspective from which biological processes may be viewed as the execution of organized information rather than merely the interaction of material components. Inspired by Martin-Löf’s constructive conception of existence and proof, a living cell may be understood not as a static object but as a continuously maintained construction. Cellular identity is not a fixed property but an active process sustained through the ongoing activity of metabolic and regulatory networks. In strongly normalizing formulations of Martin-Löf type theory, well-typed constructions reduce to normal forms. By analogy, persistent failure to reach an appropriate biological endpoint may be viewed heuristically as non-termination. Unlike the classical von Neumann architecture, in which data and execution can be conceptually separated, Martin-Löf’s constructive perspective emphasizes the inseparability of computational content from the constructions through which formal objects are generated and understood.

Recent evidence demonstrating regulated alternative RNA decoding, whereby identical RNA molecules can be constructively realized into distinct stable protein products depending on the cellular decoding context, further illustrates that biological information acquires functional meaning through its physical realization rather than through nucleotide sequence alone (Tsour et al., 2025). Although not derived from constructive type theory, such observations are conceptually consistent with a constructive view of biological information, in which information exists biologically only through the processes that instantiate it. Recent work on RNA polymerase II transcription further illustrates that cellular information is not realized by DNA sequence alone, but emerges through the coordinated interaction of promoter architecture, chromatin environment, and regulatory molecular assemblies during transcription initiation (Chen and Xu, 2026). Complementing this view, machine-learning analysis of human core promoters has identified sequence features and context-dependent interactions among promoter elements that contribute to transcriptional output (Rhyne-Carrigg et al., 2026). Together, these findings indirectly support a constructive view of cellular organization, in which biological information acquires functional meaning through its dynamic realization within a regulatory and molecular context rather than existing as an isolated sequence.

The relevance of constructive approaches to biology extends beyond a philosophical analogy. Martin-Löf’s constructive type theory provides a process-oriented framework in which formal objects are understood through explicit constructions and computational content, offering a potentially useful language for representing cellular states as dynamically generated rather than static entities. Constructive and intuitionistic approaches have been explored in the foundations of quantum mechanics (Smilga, 2017), while constructive axiomatic programmes have been developed for relativistic spacetime and quantum spacetime (Adlam et al., 2025). These examples indicate that constructivity can serve as a legitimate methodological approach to aspects of physical reality. Since cellular organization is ultimately instantiated within the same physical world, extending constructive reasoning to cellular state transitions is therefore a natural hypothesis to investigate. This does not imply that cellular systems are mathematically equivalent to constructive type theory; rather, it motivates the development of a constructive formal language capable of describing how cellular states are generated, transformed, maintained, and ultimately resolved. Within such a framework, categorical colimits could formalize the integration of compatible molecular and regulatory processes into higher-order cellular states, while coalgebraic formulations could complement this by describing their dynamic evolution and observable transitions. Together, these approaches could contribute to a constructive continuum in which cellular states are represented as continuously generated, transformed, and maintained constructions rather than as isolated static configurations. Within this continuum, progression toward a functionally appropriate terminal state would represent a biologically meaningful endpoint of the cellular trajectory, whereas persistent occupation of pathological states could correspond to failure of appropriate termination.

To make the hypothesis more than a metaphor, future formalization could represent a biological system as a constructive dynamical architecture C = (S, T, K, R), where S denotes the space of cellular states, T the admissible state transitions, K the constructive rules governing these transitions, and R the physical resources required for their realization. A transition S__i_ - > S__i+1_ would therefore represent not merely a change of state, but a physically realized constructive process governed by specific biological rules and resource constraints. Within this framework, functionally appropriate terminal states could be represented as a subset of S. Cancer would then be investigated as persistent occupation of a pathological region of state space despite available or expected transitions toward functionally appropriate terminal states.

A remarkable feature of living systems is the distinction between material turnover and organizational persistence. Molecules, proteins, and cellular components are continuously synthesized, modified, and degraded, yet the higher-order organizational patterns that define cellular identity may remain stable for years. Life therefore appears to depend not only on the persistence of material constituents but also on the preservation of informational and regulatory relationships that organize them. Entropy acts upon the physical realization of biological systems, requiring continuous energy expenditure to maintain order, whereas the organizational patterns themselves may persist despite the replacement of the material substrate through which they are instantiated.

From this perspective, biological function may depend as much on the maintenance of organizational structure as on the molecular components that realize it. The explanatory power of biological systems may therefore reside not exclusively in genes, proteins, or metabolites considered individually, but also in the network of relationships through which these components are organized and interpreted. Such a viewpoint may help explain why alterations affecting cellular organization, developmental control, or metabolic regulation can exert profound biological effects even in the absence of extensive mutational complexity.

The framework proposed here should not be viewed as a replacement for contemporary molecular oncology but as an attempt to integrate genetic, developmental, informational, and systems-level perspectives into a unified conceptual model. As advances in systems biology, artificial intelligence, and computational science continue to transform our understanding of complex systems, future progress in oncology may depend not only on identifying molecular alterations but also on understanding the organizational principles that govern how biological information is maintained, interpreted, and ultimately terminated.

## Conclusion

Cancer remains fundamentally compatible with mutation-driven evolution. The present framework proposes that, beyond mutation burden, malignant behaviour can be studied as persistent occupation of pathological biological states in which appropriate state transitions or termination fail to occur. The constructive dynamical formulation is intended as a testable theoretical direction rather than a replacement for contemporary molecular oncology.

## Data Availability

The original contributions presented in the study are included in the article/supplementary material, further inquiries can be directed to the corresponding author.
